# The causal association between polycystic ovary syndrome and susceptibility and severity of COVID-19: a bidirectional Mendelian randomization study using genetic data

**DOI:** 10.3389/fendo.2023.1229900

**Published:** 2023-09-08

**Authors:** Yu Si, Yuye Fei, Hua Ma, Yating Xu, Li Ning, Xiu Li, Qingling Ren

**Affiliations:** The Affiliated Hospital of Nanjing University of Chinese Medicine, Nanjing, China

**Keywords:** COVID-19, polycystic ovarian syndrome, Mendelian randomization, SARS-CoV-2, diabetes

## Abstract

**Introduction:**

Observational studies have reported an association between polycystic ovary syndrome (PCOS) and COVID-19, but a definitive causal relationship has not been established. This study aimed to assess this association using two-way two-sample Mendelian randomization (MR).

**Methods:**

A summary of PCOS characteristics was compiled using the PCOS summary statistics from the Apollo University of Cambridge Repository. COVID-19 susceptibility and severity statistics, including hospitalization and extremely severe disease, were obtained from genome-wide association studies from the COVID-19 Host Genetics Initiative. The primary analysis used the inverse variance-weighted method, supplemented by the weighted median, MR-Egger, and MR-PRESSO methods.

**Results:**

The forward MR analysis showed no significant impact of PCOS on COVID-19 susceptibility, hospitalization, or severity (OR = 0.983, 1.011, 1.014; 95% CI = 0.958–1.008, 0.958–1.068, 0.934–1.101; and *p* = 0.173, 0.68, 0.733; respectively). Similarly, reverse MR analysis found no evidence supporting COVID-19 phenotypes as risk or protective factors for PCOS (OR = 1.041, 0.995, 0.944; 95% CI = 0.657–1.649, 0.85–1.164, 0.843–1.058; and *p* = 0.864, 0.945, 0.323; respectively). Consequently, no significant association between any COVID-19 phenotype and PCOS was established.

**Conclusion:**

This MR study suggested that PCOS is not a causal risk factor for the susceptibility and severity of COVID-19. The associations identified in previous observational studies might be attributable to the presence of comorbidities in the patients.

## 1 Introduction

COVID-19 is a systemic disease caused by the SARS-CoV-2 virus, primarily affecting the lungs. The pathophysiological mechanisms underlying COVID-19 involve the binding of SARS-CoV-2 to angiotensin-converting enzyme 2 (ACE2) on cell membranes, triggering local and systemic inflammatory reactions, oxidative stress, and tissue hypoxia (1). These processes involve multiple organs, including the lungs, spleen, liver, heart, and kidneys. While mild cases may be asymptomatic, severe cases can lead to dyspnea/hypoxemia, acute respiratory distress syndrome, septic shock, metabolic acidosis, and multiple organ dysfunction syndrome, often culminating in death (2). The COVID-19 pandemic, a serious global epidemic and a significant public health concern, had reached unprecedented levels of incidence and mortality at the time of writing (3, 4).

PCOS is one of the most common gynecological endocrine disorders affecting women of reproductive age, with a global incidence ranging from 8% to 13% (5). The main clinical features of PCOS include hyperandrogenism, anovulation, insulin resistance, hyperinsulinemia, abnormal menstruation, and reproductive disorders. Moreover, PCOS is associated with an increased risk of developing metabolic syndrome, cardiovascular and cerebrovascular diseases, tumors, and type 2 diabetes mellitus (6).

Epidemiological research has indicated that individuals with metabolic syndrome, which encompasses conditions such as type 2 diabetes mellitus, obesity, dyslipidemia, and hypertension, are more susceptible to severe clinical outcomes of COVID-19 (7–10). Although PCOS is not explicitly implicated in these findings, it shares common complications with these metabolic conditions (11). Consequently, it is hypothesized that women with PCOS may be more vulnerable to contracting COVID-19 and experiencing severe clinical symptoms. Several small observational studies have suggested a potential predisposing role of PCOS in COVID-19. These studies have shown that compared to healthy women, those with PCOS have a 28%–50% higher likelihood of SARS-CoV-2 infection, coupled with increased incidence rates of hospitalization and mortality (12). Hyperandrogenism and chronic low-grade inflammation, which are pivotal factors in the pathogenesis of PCOS, may contribute to the progression of COVID-19 infection (13). Furthermore, COVID-19 may induce pancreatic beta-cell failure and adipocyte dysfunction, resulting in insulin resistance and potentially augmenting the risk of future PCOS development (14). However, the observed association between COVID-19 infection and PCOS in observational studies remains subject to confounding factors and the reversal of causal relationships, necessitating further investigation to establish a robust causal link between them.

MR represents a novel epidemiological method that employs genetic data to explore causal relationships between exposures and outcomes (15). By leveraging the random distribution of genetic variants during meiosis, MR helps to overcome the limitations associated with confounding and reverse causality that are commonly encountered in observational studies (16). In this study, we conducted a two-way MR analysis to elucidate the potential causal relationship between PCOS and the risk of COVID-19 infection.

## 2 Methods

### 2.1 Study design

A bidirectional two-sample MR research was conducted to evaluate the causal association between PCOS and COVID-19 susceptibility and severity. The instrumental variables employed in the analysis were chosen based on three key principles: (1) a robust correlation between genetic variation and the exposure of interest; (2) a minimal correlation between genetic variation and potential confounding factors; and (3) genetic variation that does not directly influence the outcomes under investigation (17). In this study, bidirectional MR was used to assess the effects of PCOS on COVID-19 (forward MR) and the effects of COVID-19 on PCOS (reverse MR), using the genome-wide association study (GWAS) data for PCOS and COVID-19, respectively.

### 2.2 Data sources

The data utilized in this study were derived from the COVID-19 Host Genetics Initiative GWAS round 7 meta-analyses (18). The dataset comprised 112,612 European patients with COVID-19 who were enrolled for the susceptibility phenotypic study. These patients were categorized based on laboratory-confirmed SARS-CoV-2 infection, which was identified through electronic health records (identified through the International Classification of Diseases codes or physician annotations) or self-reporting. The control group consisted of 2,474,079 individuals without confirmed COVID-19 infection. To evaluate the severity of COVID-19, two separate cohorts were utilized. The first cohort compared 24,274 hospitalized patients with 2,061,529 control patients, whereas the second cohort compared 8,779 individuals with very severe COVID-19 outcomes with 1,001,875 control individuals who were not part of the case group (source: https://www.covid19hg.org/results/r7/). Further information on the phenotypic characteristics of the study participants is presented in **Table 1**.

**Table 1:** Data sources for the analysis.

Phenotype	Source of Genetic Variants	
	Consortium	Participants
Polycystic ovarian syndrome	–	Cases: 10,074 participants diagnosed with PCOS.
		Controls: 103,164 individuals not diagnosed with PCOS.
COVID-19 susceptibility	Susceptibility	Cases: 112,612 participants who were verified as COVID-19 through laboratory testing for SARS-CoV-2 infection, electrical health records, or self-reporting.
		Controls: 2,474,079 participants who were a part of the cohorts but were not counted as cases.
COVID-19 severity	Hospitalized	Cases: 9,986 patients with COVID-19 were hospitalized.
		Controls: 1,877,672 participants who were excluded from the analysis.
	Very severe disease	Cases: There were 8,779 individuals classified as very severe patients who either passed away or needed breathing assistance Controls: 1,001,875 individuals who were excluded from the analysis and served.

A comprehensive analysis of statistical data on PCOS was conducted, utilizing data obtained from the authoritative repository available at https://www.repository.cam.ac.uk/items/3ccbf35f-5f69-4b46-b4fc-12cb67af71ae. The summary encompasses the findings of a GWAS comprising seven independent cohorts (19). The PCOS cohort consisted of 10,074 individuals diagnosed with PCOS, whereas the control group comprised 103,164 healthy female participants. To ensure accuracy, the statistical analysis accounted for variables such as age, age squared, and gender. For further information regarding the characteristics of the seven independent cohorts, interested readers can refer to the original article (19). Information pertaining to ethical approval and consent for data utilization was obtained by referencing the original article.

### 2.3 Selection of genetic instruments

To ensure the reliability of the analysis and minimize potential statistical biases that could originate from the original GWAS, rigorous criteria were applied during the selection of genetic instruments. Specifically, only single nucleotide polymorphisms (SNPs) with a significance threshold of *p* < 5 × 10^−8^ were considered for inclusion. Subsequently, only SNPs exhibiting linkage imbalance (R^2^ < 0.01) and clustering within genomic regions separated by at least 10 Mb were retained for further analysis.

Utilizing the PhenoScanner database (http://www.phenoscanner.medschl.cam.ac.uk/), several instrumental variables related to other phenotypes that could potentially influence the outcomes were identified. These variables included rs9264740 (associated with diabetes mellitus diagnosed by a doctor, self-reported type 1 diabetes, and treatment with insulin product), rs1128175 (associated with diabetes diagnosed by a doctor; medication for cholesterol, blood pressure, or diabetes mellitus: insulin, started insulin within one year of diagnosis of diabetes mellitus, treatment with insulin product), rs550057 (associated with type 2 diabetes mellitus; medication for cholesterol, blood pressure, or diabetes mellitus: cholesterol-lowering medication), rs1498399 (associated with body mass index and weight), and rs1634761 (associated with weight). These selected SNPs for exposure to COVID-19 were found to be significantly correlated with diabetes phenotypes (e.g., diagnosed with diabetes mellitus, history of insulin therapy) and body mass phenotypes (e.g., weight, body mass index). Noteworthy studies have indicated that diabetes mellitus represents a prominent risk factor for susceptibility to and severity of SARS-CoV-2 infection. Compared to non-diabetic patients, patients with diabetes mellitus who contract SARS-CoV-2 exhibit elevated IL-6 and CRP levels. This phenomenon might be attributed to the inherent proinflammatory effect of diabetes mellitus, which may contribute to the systemic inflammatory response observed in COVID-19 (20). Moreover, patients with diabetes mellitus experience prolonged hospital stays, more severe pneumonia symptoms, and higher clinical mortality rates (21). Recent evidence further suggests that SARS-CoV-2 can directly induce acute or chronic damage to the pancreas, thereby influencing the regulation of glucose metabolism and insulin sensitivity, and even potentially inducing diabetes mellitus in individuals without prior diabetic conditions (22). Meanwhile, inflammation and the immune system in obese individuals could play a role in relation to viral diseases. Adipose tissue produces pro-inflammatory cytokines in high amounts, causing chronic low-grade inflammation and immune dysregulation (23). Consequently, these instrumental variables were excluded both before and after conducting the MR analysis.

The coefficient of determination (R^2^) reflects the potential of genetic factors to account for variations in exposure scenarios. Additionally, F statistics (F > 10) were utilized to ensure the inclusion of robust instrumental variables while excluding weaker ones. Comprehensive details regarding the chosen SNP are available in the **Supplementary Document**.

### 2.4 MR analysis

Data on PCOS were analyzed using a two-sample MR approach. The primary method employed in this study was the inverse-variance weighted (IVW) method (24), followed by MR Egger and weighted median as secondary methods (25). It is important to note that the MR Egger method typically yields larger standard errors of causal estimation and lower causal effect estimates compared to IVW (26). Thus, IVW was utilized to investigate the causal link between exposure and outcomes, and the findings were presented as ORs with corresponding 95% confidence intervals (CIs). Additionally, three sensitive assessments were performed, including weighted median and MR-Egger methods, to assess the impact of different assumptions and investigate potential pleiotropy-induced biases (25, 27, 28). If more than 50% of the instrumental variables are reliable, the weighted median approach determines the median of the empirical distribution of MR estimates, providing trustworthy estimates (25).

**Figure 1:**
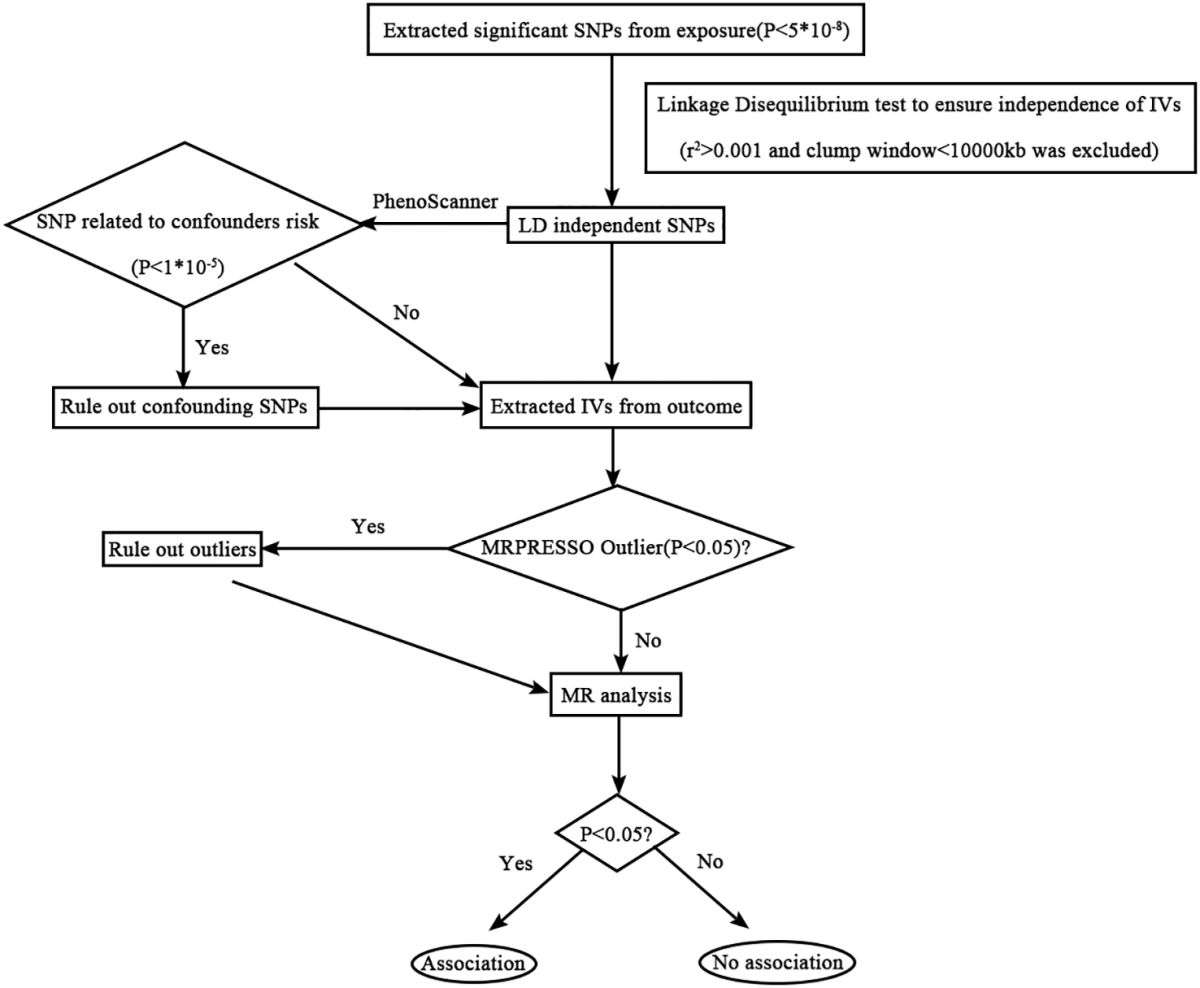
Flowchart detailing the analytical techniques and the step-by-step process of the MR analysis.

Several other sensitivity analyses were performed to evaluate and address the potential sources of bias in the study. Cochran’s Q-test was used to assess heterogeneity among the instrumental variables, whereas MR-PRESSO was employed to detect and correct violations of the instrumental variable assumptions (28). MR-PRESSO is particularly useful when the horizontal pleiotropic effects account for less than 10% of the total variation (29). Leave-one-out analysis was utilized to examine the influence of individual SNPs on the MR findings. Additionally, the statistical power of the studies was assessed using the website (http://glimmer.rstudio.com/kn3in/mrnd/) (30). A flowchart illustrating the step-by-step process of the MR analysis is presented in **Figure 1**.

This study utilized the two-sample MR and MR-PRESSO software packages for conducting the MR analysis. All research involving statistics was conducted using R software (version 4.2.1), and STATA 12.0 and R software were combined to visualize the data.

## 3 Results

### 3.1 PCOS and COVID-19: a causal link

**Figure 2:**
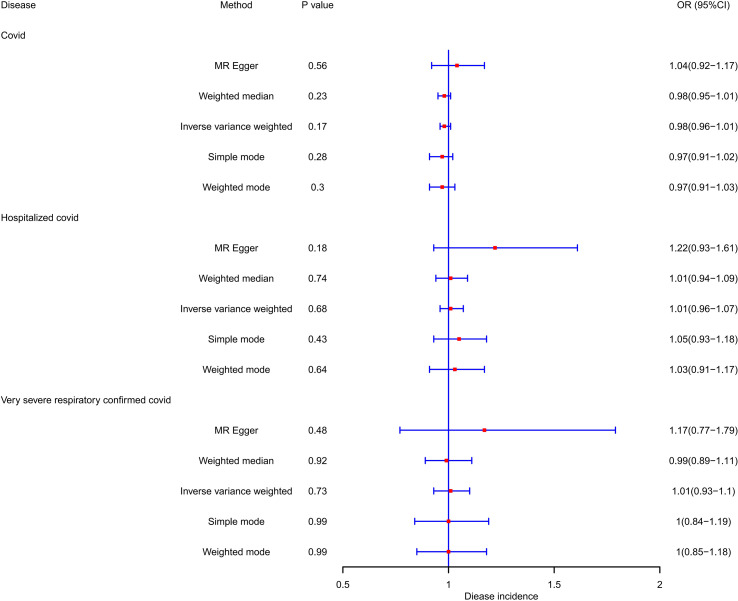
OR and 95% CI of the causal relationship between PCOS and the risk of COVID-19.

PCOS was neither a risk factor nor a protective factor in the susceptibility to COVID-19, hospitalization, or a severe disease phenotype. The IVW method yielded an OR of 0.983 (95% CI = 0.958–1.008, *p* = 0.173) for susceptibility to COVID-19, an OR of 1.011 (95% CI = 0.958–1.068, *p* = 0.68) for hospitalization, and an OR of 1.014 (95% CI = 0.934–1.101, *p* = 0.733) for a severe disease phenotype. Furthermore, evaluations using the Q test, MR Egger intercept, and MR-PRESSO showed no notable heterogeneity, pleiotropy level, or outliers linking PCOS to the risk of COVID-19. **Table 2** and **Figure 2** demonstrate the comprehensive outcomes of the various MR analyses. Scatter plots, funnel plots, and leave-one-out analyses are available in the **supplementary documents**.

**Table 2:** MR-derived evaluation of the causal impact of PCOS on COVID-19.

	Outcome	nSNP	IVW		Weighted Median		MR-Egger	
			OR (95%CI)	*P*	OR (95%CI)	*P*	OR (95%CI)	*P*
PCOS	COVID-19 susceptibility	12	0.983 (0.958,1.008)	0.173	0.980 (0.949,1.013)	0.231	1.039 (0.918,1.175)	0.562
	COVID-19 hospitalization	12	1.011 (0.958,1.068)	0.684	1.012 (0.940,1.090)	0.743	1.223 (0.931,1.606)	0.178
	COVID-19 severity	12	1.014 (0.934,1.101)	0.733	0.995 (0.892,1.110)	0.925	1.174 (0.768,1.795)	0.476

### 3.2 COVID-19 and PCOS: a causal link

Likewise, our investigation found no supportive evidence indicating that susceptibility to COVID-19, hospitalization, or the manifestation of a severe disease phenotype has a significant impact on the risk or protection against PCOS. Employing the IVW method, the ORs were estimated as 1.041 (95% CI = 0.657–1.649, *p* = 0.864) for susceptibility to PCOS, 0.995 (95% CI = 0.85–1.164, *p* = 0.945) for hospitalization, and 0.944 (95% CI = 0.843–1.058, *p* = 0.323) for very severe disease phenotype due to COVID-19. None of these associations reached statistical significance. During the analysis, we identified instrumental variables such as rs9264740, rs1128175, rs550057, rs1498399, and rs1634761, which were significantly associated with diabetes mellitus and body mass which may act as confounding factors in the COVID-19 phenotype. To address this concern, these instrumental variables were excluded from the analysis, and the MR analysis was re-evaluated, leading to consistent conclusions with the initial analysis.

**Figure 3:**
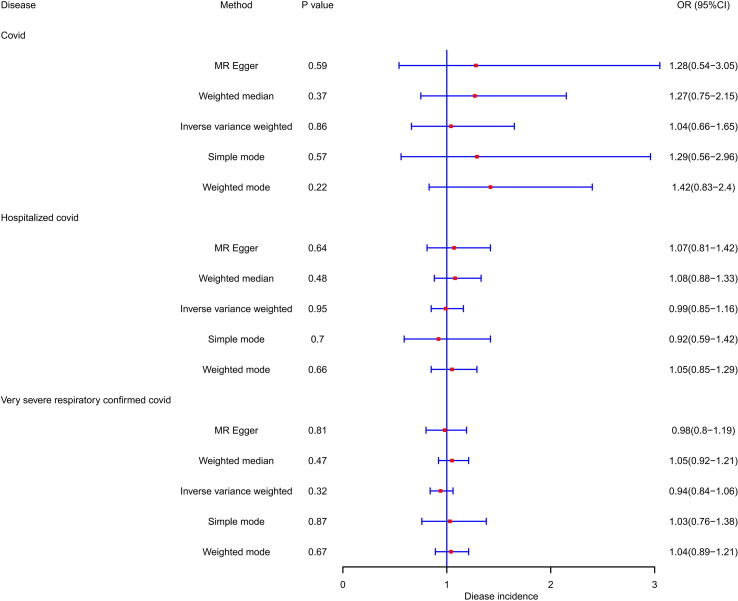
OR and 95% CI of the causal relationship between the risk of COVID-19 and PCOS.

Furthermore, our study revealed that none of the COVID-19 phenotypes emerged as protective factors for PCOS (*p* > 0.05). To mitigate the potential heterogeneity in research findings, we employed the IVW random effects approach. The comprehensive results of this analysis are shown in **Table 3** and **Figure 3**. Scatter plots, funnel plots, and leave-one-out analyses are available in the **Supplementary Documents**.

**Table 3:** MR-derived estimates of the causal influence of COVID-19 on PCOS.

	Outcome	nSNP	IVW		Weighted Median		MR-Egger	
			OR (95%CI)	*P*	OR (95%CI)	*P*	OR (95%CI)	*P*
COVID-19 susceptibility	PCOS	13	1.041 (0.657,1.649)	0.864	1.271 (0.752,2.145)	0.370	1.282 (0.538,3.055)	0.586
COVID-19 hospitalization		30	0.995 (0.850,1.164)	0.945	1.078 (0.875,1.328)	0.480	1.072 (0.807,1.424)	0.636
COVID-19 severity		25	0.944 (0.843,1.058)	0.323	1.052 (0.917,1.206)	0.471	0.975 (0.796,1.945)	0.810

## 4 Discussion

COVID-19, which has been declared by the WHO as a global public health epidemic, has become one of the most concerning diseases in recent years. As of September 26, 2022, there were nearly 620 million laboratory-reported cases of COVID-19 worldwide, with more than 6.5 million deaths (https://www.worldometers.info/coronavirus/). Although COVID-19 can affect people of all ages and backgrounds, patients with pre-existing medical conditions are at an increased risk of experiencing severe outcomes and increased mortality rates (31, 32).

Our bidirectional two-sample MR study incorporated data from two distinct consortiums, particularly focusing on individuals of European ancestry. To ensure the reliability and accuracy of our genetic instruments, a meticulous screening process was conducted utilizing the PhenoScanner database.

Large sample size and a homogeneous study population are the prerequisites for the validity of the MR analysis results, and the assumptions of association, independence, and exclusion should be met to obtain valid causal inferences between the exposure and outcome variables.

In conclusion, our extensive MR analysis indicated no evidence of a causal relationship between PCOS and susceptibility to or severity of COVID-19. The results suggest that PCOS has minimal to no effect on the likelihood or severity of COVID-19 infection.

Previous observational studies have suggested a potential association between PCOS and COVID-19, which may be influenced by confounding factors and reverse causality. A closed cohort study conducted in the British population (12) aimed to investigate this relationship. The study included 21,292 women diagnosed with PCOS and randomly selected 78,310 healthy women. The incidence rate of COVID-19 was higher in women diagnosed with PCOS, at 18.1 per 1000 person-years, compared to women without PCOS, at 11.9 per 1000 person-years.

Findings from basic research indicate that androgens may contribute to the progression of COVID-19 by modifying androgen-mediated immune control and upregulating the expression of TMPRSS2, a cellular co-receptor required for SARS-CoV-2 infection (33). Immune dysfunction and a persistent inflammatory state are brought on by the endocrine–immune axis of patients with PCOS. The compensatory hyperglycemia, hyperandrogenism, and insulin resistance associated with PCOS may render individuals more susceptible to COVID-19 (13, 34, 35). Additionally, PCOS may increase susceptibility to COVID-19 through comorbidities such as obesity (36, 37). Notably, a study revealed significantly increased mRNA expression of ACE2 and TMPRSS2, key molecules involved in SARS-CoV-2 cell entry, in the livers of patients with advanced nonalcoholic fatty liver disease (NAFLD) (38).

Owing to the existence of conflicting findings among studies, not all studies have reached a consistent conclusion regarding the association between PCOS and the susceptibility to and severity of COVID-19. One such study conducted by HIPAA Limited, a subsidiary of the University of California Coronavirus Disease Research Dataset (UC CORDS), utilized health records and statistical analyses of patients undergoing COVID-19 testing at the University of California medical institutions. The results of this study did not provide evidence to support an increased risk of COVID-19 infection, hospitalization, or mortality among women with acne vulgaris, PCOS, or hirsutism. Consequently, establishing a causal relationship between PCOS and susceptibility to and severity of COVID-19 based solely on observational studies is challenging.

Although our study did not yield positive results, we formulated certain assumptions to understand the relationship between PCOS and COVID-19. Notably, PCOS is influenced by both genetic and environmental factors and is associated with comorbidities such as obesity, insulin resistance, diabetes mellitus, NAFLD, cardiovascular diseases, and cerebrovascular diseases. These comorbidities are established risk factors for COVID-19 susceptibility and severity, suggesting their potential role in the pathogenesis of COVID-19. The interaction between PCOS and COVID-19 involves a complex interplay of comorbidities rather than a straightforward causal relationship. The use of instrumental variables in MR analysis to control for confounding factors, such as diabetes mellitus and body mass phenotype, is a robust approach to strengthen the validity of the findings. By excluding these variables from the analysis, we aimed to minimize potential biases and ensure a more accurate assessment of the potential causal relationship between PCOS and COVID-19 infection. While the study did not establish a direct causal relationship between PCOS and COVID-19 infection and severity, it highlights the importance of considering the broader context of comorbidities and underlying risk factors that may influence disease outcomes.

MR analysis aims to minimize the impact of confounding variables to the greatest extent possible. Furthermore, we employed an extensive collection of independent datasets to secure genetic instruments, thereby diminishing the probability of bias introduced by a limited number of cases. However, given that our study exclusively focuses on individuals of European ancestry, there are inherent limitations to our conclusions, highlighting the importance of expanding the sample study to other populations.

Although MR studies are valuable tools for investigating causal relationships between exposures and outcomes using genetic variants as instrumental variables, they cannot account for other non-genetic factors, such as lifestyle and environmental factors, which may also play a role in the observed association. Therefore, this conclusion should be interpreted with caution, and further research is needed to fully understand the causal relationship between PCOS and COVID-19.

## 5 Conclusion

In conclusion, MR analysis does not provide evidence supporting PCOS as a causal risk factor influencing the susceptibility or severity of COVID-19. The previously observed correlation between PCOS and COVID-19 may be attributed to the influence of comorbidity factors. These comorbidities, such as obesity, insulin resistance, diabetes, and other cardiovascular and metabolic conditions, rather than PCOS itself, could be contributing to the association observed in these studies.

## Data availability statement

The original contributions presented in the study are included in the article/**Supplementary Material**. Further inquiries can be directed to the corresponding author.

## Author contributions

The study was designed by YS and YF. Data analysis was conducted by YS, XL, and LN. YS, HM, and YX contributed significantly to the inception of the project and the manuscript. QR provided revisions and approved the paper. YS and YF made equal contributions to this article. All authors contributed to the article and approved the submitted version.
