# Supplementary figures and images for: The causal association between polycystic ovary syndrome and susceptibility and severity of COVID-19: a bidirectional Mendelian randomization study using genetic data
Source: Front Endocrinol (Lausanne). 2023 Sep 8;14:1229900. doi: 10.3389/fendo.2023.1229900 (PMC10515223; doi:10.3389/fendo.2023.1229900)

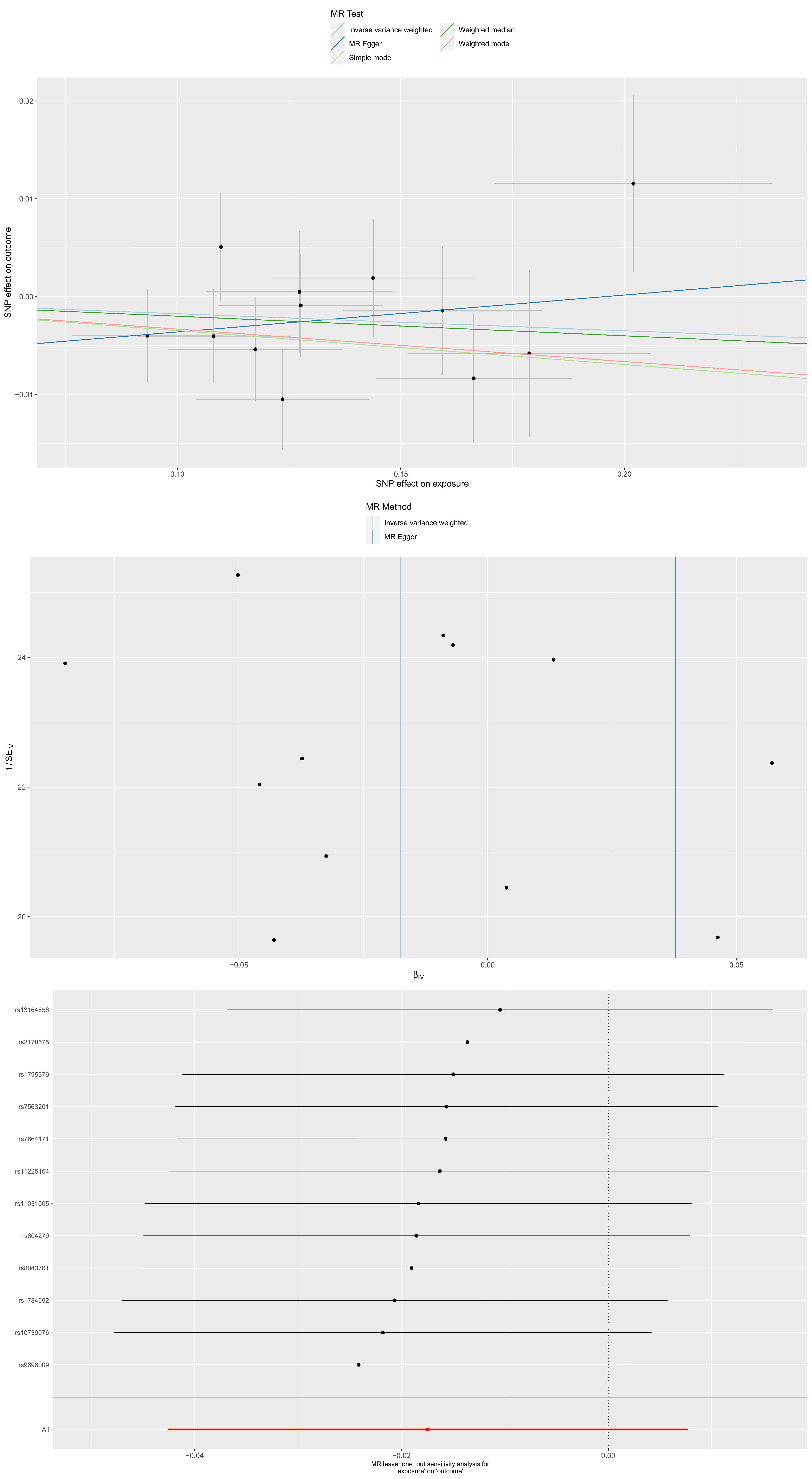

Supplement: Supplementary file 1 [file Image_1.jpeg]

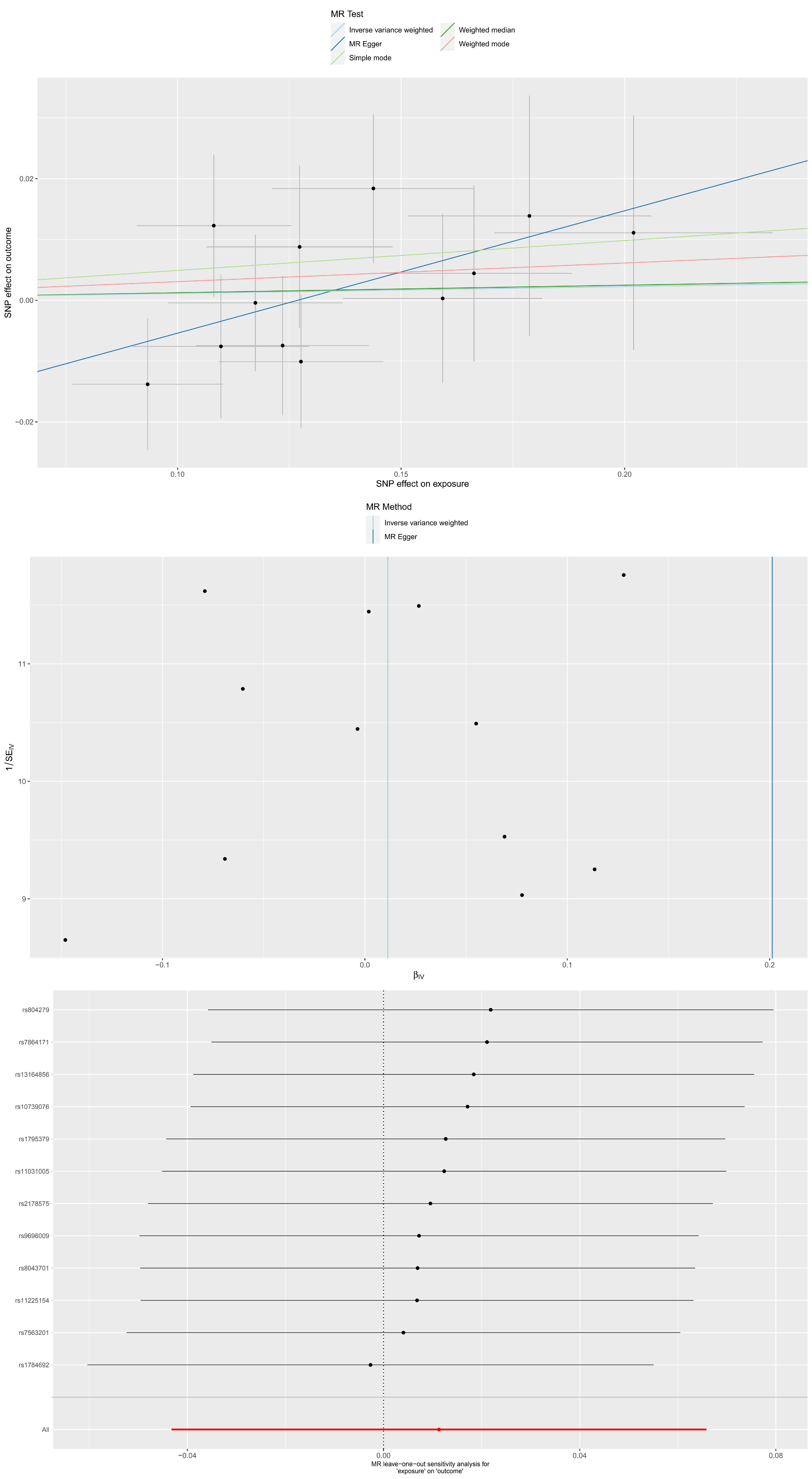

Supplement: Supplementary file 2 [file Image_2.jpeg]

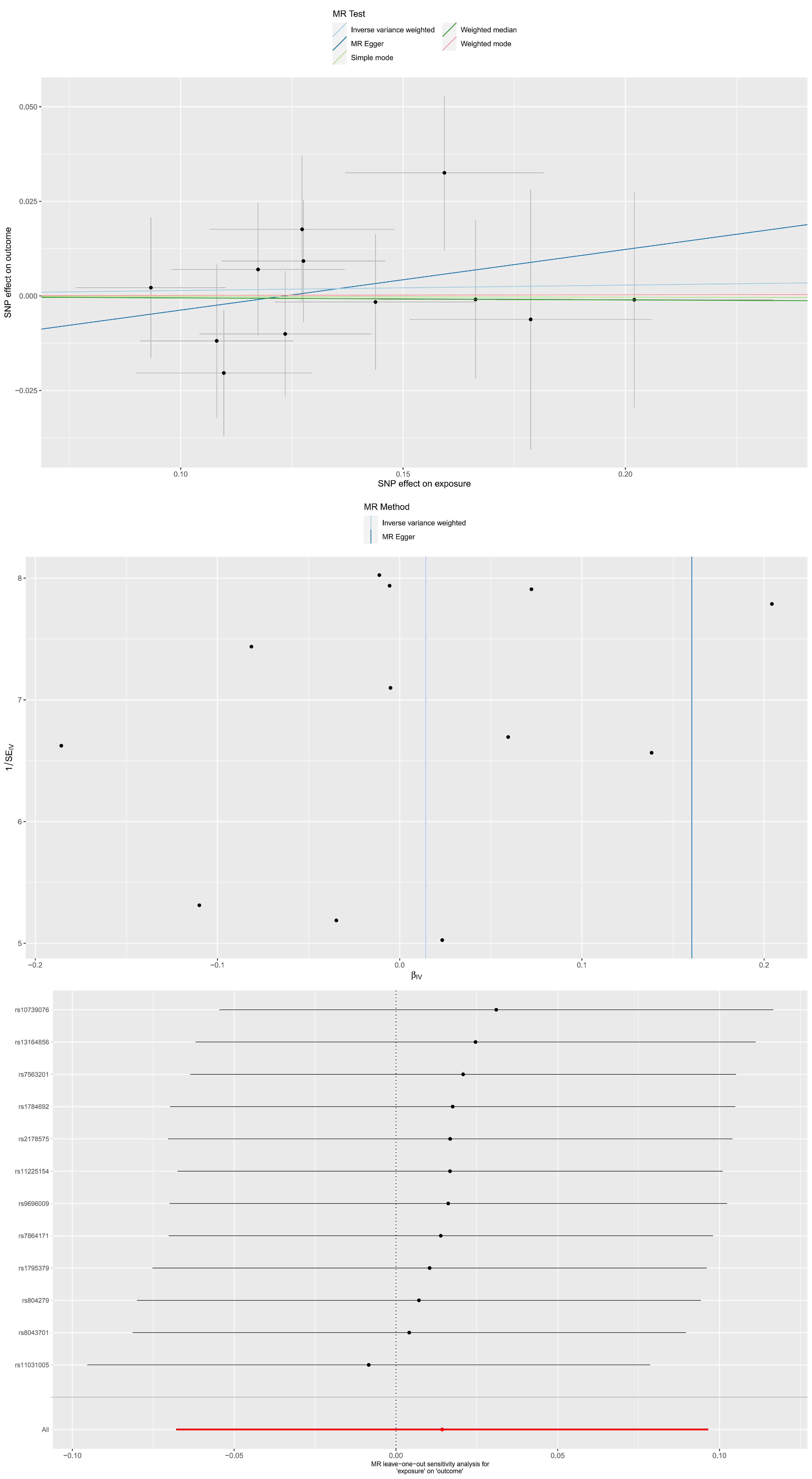

Supplement: Supplementary file 3 [file Image_3.jpeg]

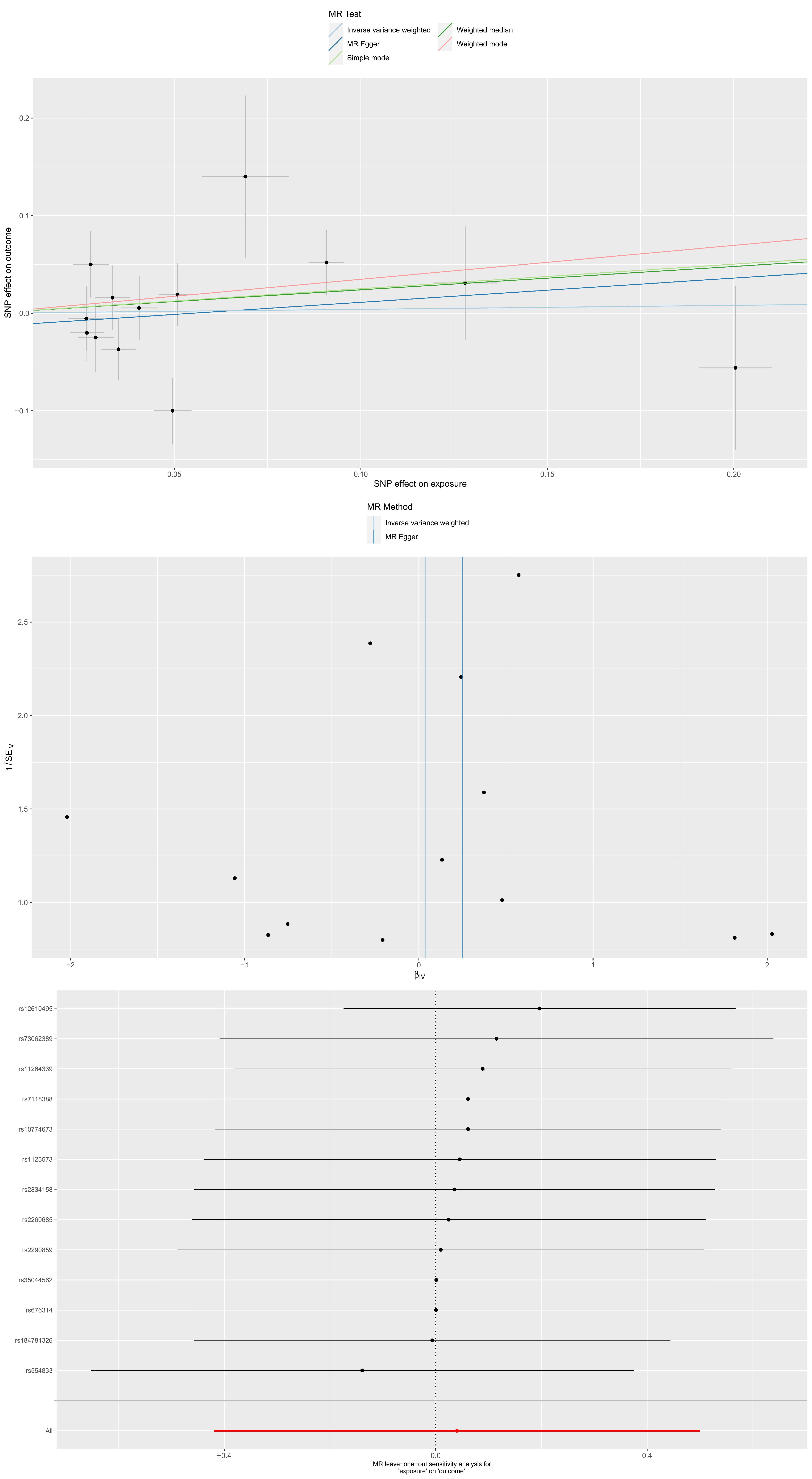

Supplement: Supplementary file 4 [file Image_4.jpeg]

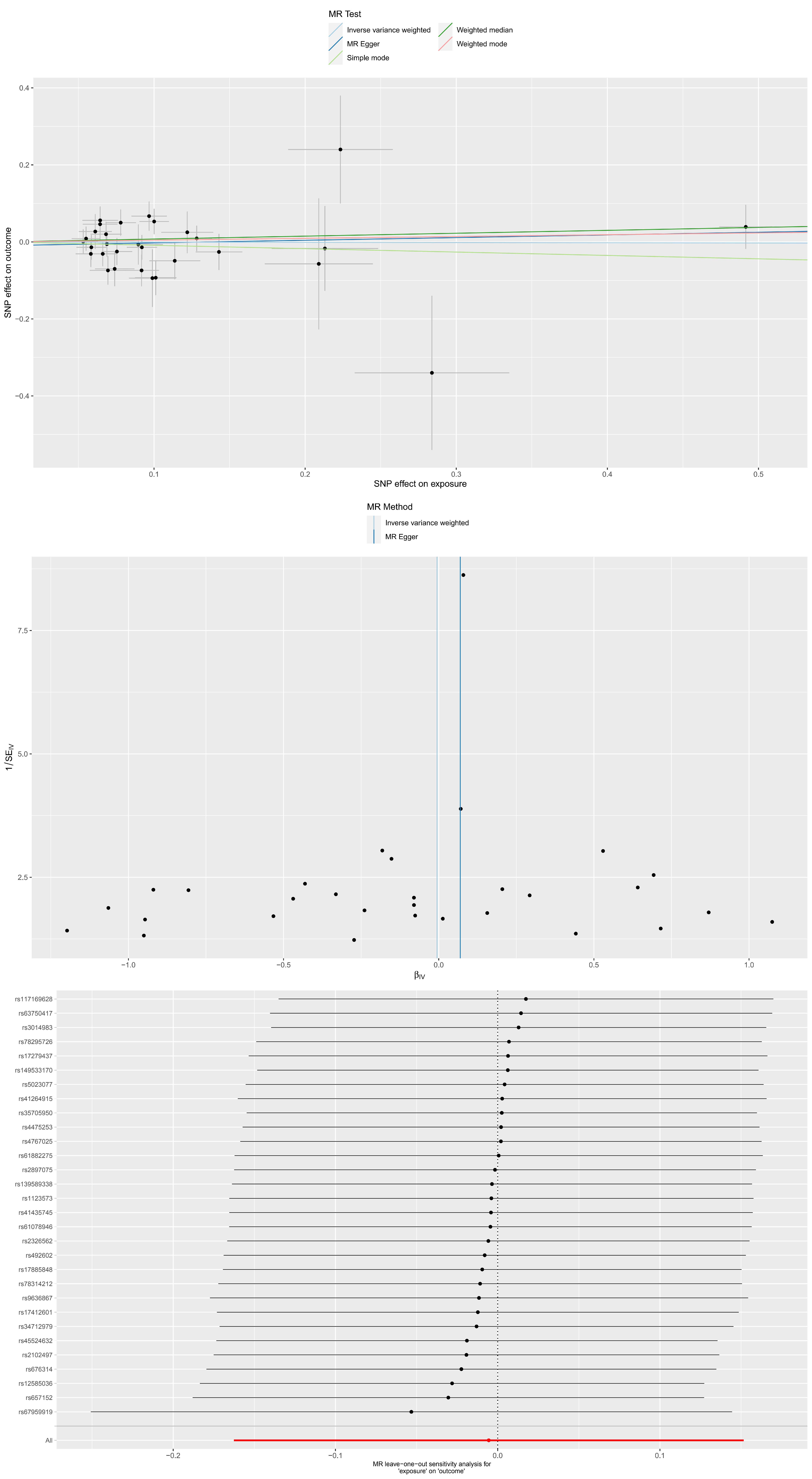

Supplement: Supplementary file 5 [file Image_5.jpeg]

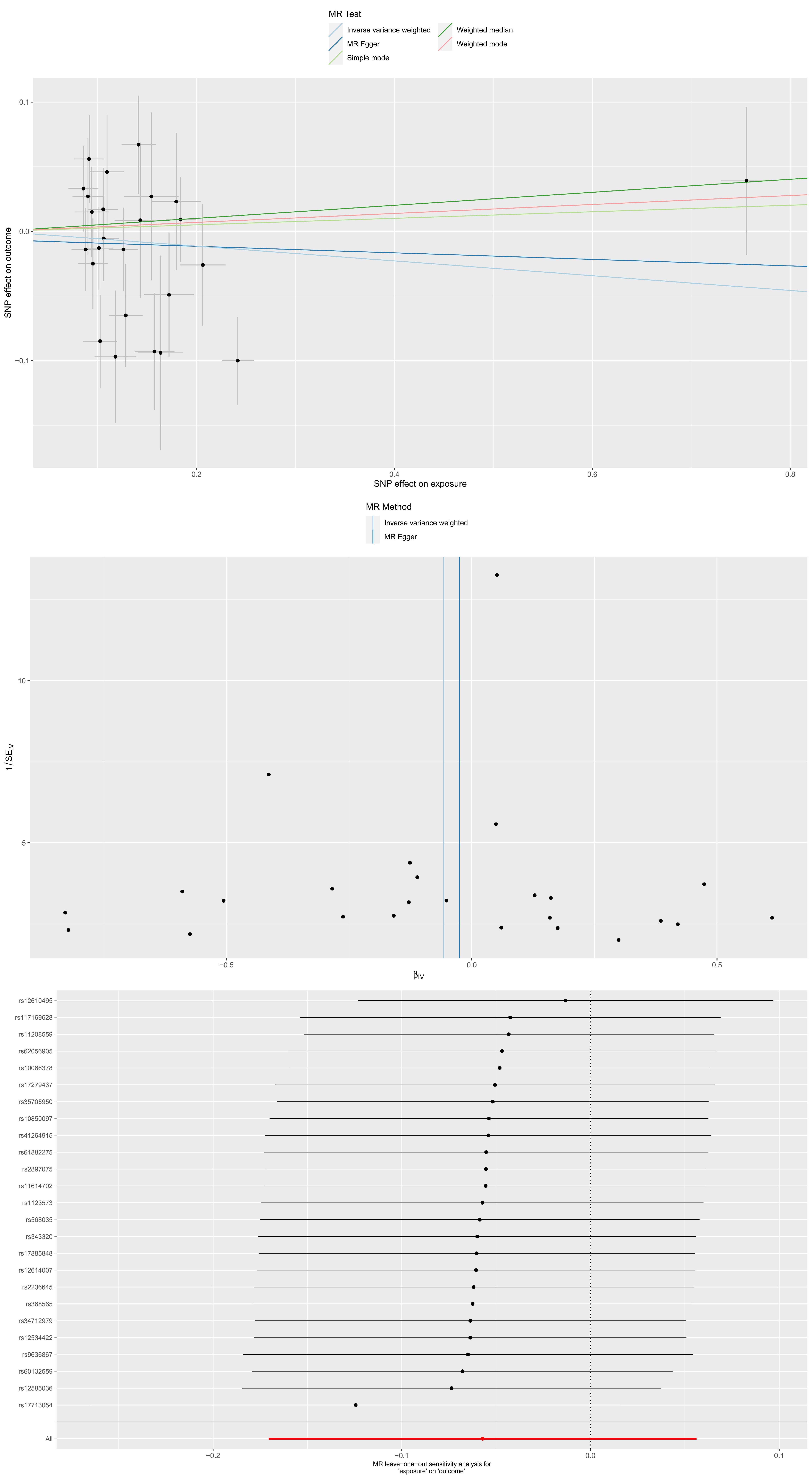

Supplement: Supplementary file 6 [file Image_6.jpeg]
